# What Can We Learn from Medical Whistleblowers?

**DOI:** 10.1371/journal.pmed.0020209

**Published:** 2005-05-27

**Authors:** Jeanne Lenzer

## Abstract

High-profile medical whistleblowers tell their stories of how American medicine's close ties with the pharmaceutical industry may be putting patients' safety at risk.

A year ago, I received an E-mail from a research scientist at a major pharmaceutical company. The scientist had read my articles on whistleblowers who had raised concerns about the undue influence of the pharmaceutical industry on American medicine My industry source had information for me about drug company practices, but—out of fear of career ruin—would only talk on the condition that I would conceal the scientist's identity.

For the next year or so, I had repeated contacts with the scientist. As I listened to this researcher—and to the other medical whistleblowers that I continued to interview—it occurred to me that each whistleblower was like the proverbial blind man with a hand on the elephant. Each could describe one piece of the puzzle, but the full picture could only emerge by bringing these whistleblowers together.

With an eye to focusing on the systemic problems that have allowed American medicine to be unduly influenced by industry, on May 15, 2005, I brought together five whistleblowers in Washington, D. C. I asked them each to tell their story and to suggest ways to restore objectivity to medicine and medical research.

## The Whistleblowers

Four whistleblowers attended in person, and the anonymous industry scientist participated via speakerphone. The whistleblowers came from an extraordinary variety of different professional backgrounds.

### 

#### David Graham

This Food and Drug Administration (FDA) safety officer raised concerns about the cardiovascular side effects of rofecoxib (Vioxx) and other Cox-2 inhibitors. He testified at a United States Senate Finance Committee hearing on rofexocib, the FDA, and Merck [1,2]. Graham attended the roundtable in his own personal capacity and was not representing the FDA.

Each whistleblower was like the proverbial blind man with a hand on the elephant.

### 

#### Allen Jones

This investigator at the Pennsylvania Office of the Inspector General led an investigation into an off-the-books account, funded in part by drug companies, from which payments were made to state employees to develop a medication treatment algorithm. He filed a civil rights lawsuit against the Pennsylvania Office of the Inspector General to protect his right to publicly discuss his findings, and was later fired from his job for talking to the press [3–6].

### 

#### Stefan Kruszewski

This Harvard-trained psychiatrist was hired by the Bureau of Program Integrity in the Pennsylvania Department of Public Welfare to oversee the state's mental health and substance misuse programs. He filed a law suit in a federal court in the Middle District of Pennsylvania, charging that he was fired after uncovering widespread abuse and fraud in the bureau [7,8].

### 

#### Kathleen Slattery-Moschkau

This former drug representative left the pharmaceutical industry after witnessing marketing practices that she found disturbing. She wrote and directed the movie *Side Effects*, a fictionalized account of her experiences [9,10].

### 

#### The anonymous research scientist

This is an industry insider who said to me, ahead of the roundtable, that the culture of secrecy at drug companies too often results in claims that are closer to “propaganda” than science.

## Lessons Learned from the Roundtable

### 

#### Ties between drug regulators and industry may influence new drug approval

David Graham described the frustrations he had felt in his almost 20 years of experience as an FDA drug safety officer. Although he was instrumental, he said, in getting ten drugs off the market because of safety concerns, his experience was like a salmon swimming upstream—“a single individual…against the tide.” The tide, he said, “is an entire institution whose mission is to approve drugs and make industry happy.”

The FDA, said Graham, is in a “collaborative relationship” with industry. The FDA gets money from drug companies through the Prescription Drug User Fee Act of 1992 (see http://www.fda.gov/cber/pdufa.htm) “to approve new drugs and approve them more quickly.” The mindset at the FDA, he said, is that “we will find a reason to approve a drug no matter how small the indication for the drug.” Graham explained that a senior official at the FDA had told him: “industry is our client.“

When the FDA knows there is a serious problem with a new drug, he said, the FDA deals with this by saying, “well, we'll handle it in labeling” even though, said Graham, “FDA knows labeling doesn't work.”

“There is no independent voice for drug safety in the United States,” he said. The upper-level managers in the FDA's Office of Drug Safety are appointed from the FDA's Office of New Drugs, which approves new medicines. This makes the Office of Drug Safety “captive,” he said, to the Office of New Drugs.

The anonymous scientist said that in order to speed up drug approval, companies “don't measure things like whether we are really curing the disease, or prolonging life, or preventing hospitalization, or whether a patient is truly more functional. Oftentimes, we're measuring intermediate, lesser things, markers, predictors—we *hope*—of these clinical endpoints, but they may or may not be accurate.”

And the FDA, said the scientist, requires just two positive studies to grant approval to a new drug, but there is no limitation on how many negative studies can be done before one or two positive studies are produced. This can lead to approval of a drug even when most studies are negative or show no effect.

Both Graham and the anonymous scientist suggested putting an end to the Prescription Drug User Fee Act, and Graham argued that there needs to be independent authority for those in charge of drug safety. They indicated that two bills in Congress, introduced by Senator Grassley and by Congressman Hinchey, at least partly address these concerns.

“The pharma–FDA complex has to be dismantled,” said Graham, “and the American people have to insist on that, otherwise we're going to have disasters like Vioxx that happen in the future.”

### 

#### The race to approve new drugs without proper safety testing may be compromising the public's health

“Drug companies assiduously avoid acquiring information about side effects,” said the industry scientist. “Drug companies will not conduct safety studies unless they have to—meaning basically that they're required by a regulator—and that rarely happens.” High-risk patients who might have a bad reaction to a drug, said the scientist, “are excluded from studies deliberately, even though, when the drug is approved, these patients will be targeted for sales.” When a safety study is proposed within the industry, said the scientist, “a typical response will be that if we conducted a study to find out if there was a safety problem, people would learn about it and think we *had* a problem [which] would destroy the image of safety that has been so carefully constructed.”

“There is no independent voice for drug safety in the United States.”

Studies are too small and are conducted over too brief a period to properly assess safety: “The largest studies—the phase three studies, [which] might be several thousand people—last for a few months. If drugs kill one in several thousand per year, this would be a public health catastrophe. A blockbuster drug with that kind of hazard associated with it could be associated with tens of thousands of deaths a year, and it would never be detected in studies of the kind that we routinely submit and are the basis for approval.” These drugs, said the scientist, and these kinds of risks, are “essentially out there now, unlabeled, unnoticed, all beneath the radar.”

The scientist said that, “to ensure that safety problems will go unnoticed, we compound the problem of conducting small studies by setting a statistical threshold for acknowledging the safety problem that is so high that you know in advance it could never be reached for any serious side effect, like myocardial infarction.” This practice, said the scientist, “virtually ensures that if a bad side effect happens to show up, it's not going to reach the arbitrary level that we call statistically significant, and the company can maintain that it's just bad luck.” And if a bad result does happen, “typically a company is not going to publish the study at all. If they do publish it, the bad result can be omitted as ‘not statistically important.’”

### 

#### The funding of state officials by industry may be affecting prescribing patterns

Allen Jones described how he believed that drug companies were acting at the state level to influence the prescribing of psychiatric medications.

“I began to investigate an account into which pharmaceutical companies were paying money that was being accessed by state employees,” he said. “Additionally, I found that various pharmaceutical companies were paying state employees directly—also giving them trips, perks, lavish meals, transportation, honorariums up to $2,000 for speaking in their official capacities at drug company events. They were given unrestricted educational grants that were deposited into an off-the-books account—unregistered, unmonitored, literally operated out of a drawer.”

These same state officials, he said, were responsible for dictating clinical policy and writing guidelines for the treatment of patients in the state system. These officials were, he said, receiving money from companies with a stake in the guidelines. “The protocol they [the officials] were developing was called the Texas Medication Algorithm Project, TMAP, which began in Texas in the mid-90s. It outlined detailed medication guidelines for schizophrenia, depression, and bipolar disorder. It recommends almost exclusive usage of newer, patented, very expensive atypical antipsychotics, SSRIs [selective serotonin uptake inhibitors], and mood stabilizers.” The Texas Medication Algorithm Project, said Jones, was based on “expert consensus” from industry-supported meetings.

Jones said that when he wanted to investigate these findings, he was shut down. “I was told point black, ‘Look, drug companies write checks to politicians, they write checks to politicians on both sides of the aisle—back off.’” He was told, he said, to “quit being a salmon, quit swimming against a stream.” He wouldn't back down from his investigation, he said, and was demoted. On November 22, 2002, he filed a civil rights lawsuit “to preserve my job and my right to speak out.” His employer, he said, took him off investigative duties altogether.

Stefan Kruszewski, who has filed a law suit in a federal court in Pennsylvania, raised concerns to his seniors in the Pennsylvania Department of Public Welfare about prescribing practices in the state that he did not feel were evidence based, and said he lost his job for raising his concerns. For example, he alerted his seniors to the off-label prescribing of the anticonvulsant gabapentin (Neurontin) for mood disorders and addictive disorders.

“The pharmaceutical industry is the single most powerful lobbying group on Capitol Hill—outspending even the oil and banking industries,” said Jones. “It should come as no surprise that the ties go far beyond just the mental health officials who wrote the guidelines, but extend to many of the politicians who, in the end, allowed an investigation into pharma corruption to be dropped, and the investigator—me—to be fired.”

Efforts to detect and deter fraud and abuse due to these conflicts, he said, “will be likely to be undermined as long as those charged with detecting fraud and abuse, like the [Pennsylvania] Inspector General, are appointed by politicians who are themselves beholden to the drug industry. Such positions should instead be filled by career civil servants and not political appointees.”

### 

#### Regulatory agencies are not being held accountable

In comments that echoed his testimony to the US Senate Finance Committee, Graham said that, “FDA was the single greatest obstacle to doing anything effective with Vioxx. As a result, nearly 60,000 people probably died from that drug. That's as many of our soldiers that were killed in the Vietnam war [who] died as a result of Vioxx use. And FDA had the opportunity, the responsibility, to stop that and didn't. In fact, FDA allowed it to continue. In my book, FDA shares in the responsibility for those deaths and yet it's not being held accountable by Congress.” Congress itself, added Graham, is deeply beholden to the drug industry since many politicians receive “often quite a bit of campaign contributions” from the industry.

Kruszewski reflected upon the problems he said he had encountered in Pennsylvania, saying that “there is no accountability in the system for oversight [agencies].” He has become “a stronger advocate than ever for a federal patient bill of rights.”

### 

#### Marketing departments can influence doctors’ prescribing habits

The research scientist said that the job was attractive because of the “many excellent drugs” developed, such as drugs to treat HIV, but the scientist “also saw drugs marketed in a way that will exaggerate the benefits and conceal the risks.”

Kathleen Slattery-Moschkau gave an insider's view of drug marketing practices, from her former experiences as a drug rep. She clutched her head in disbelief as she told the roundtable that doctors would come up to her with patients' charts asking her for advice on treating patients. Slattery-Moschkau, like most of the drug representatives she came to know over the years, had no science background at all.

“Drug companies assiduously avoid acquiring information about side effects.”

The various techniques drug representatives were trained in to “educate doctors” eventually proved to be not just “comical” but “also scary,” she said. “Whether it was hiring, training, what we were told to say about drugs and what we were told not to say,” it was marketing, not science, that dominated. One of the techniques used by drug companies was to buy doctors' prescribing records so drug representatives knew “to the dime” what drugs doctors were prescribing and could tailor their marketing to them. Drug representatives developed “personality profiles” on doctors and were taught to pitch their sales to specific personality types. Representatives were compensated, she said, by “how many prescriptions we could encourage.”

Both Slattery-Moschkau and the industry scientist described tensions within drug companies between marketing departments and industry scientists. “The marketing spin on things,” said the scientist, “carries the day.”

### 

#### The published medical literature contains many biases

“When studies are published,” said the scientist, “they are frequently written not by the trained research scientist, who might have designed and analyzed the study, but by a designated medical writer with little if any background in research, but who is trained instead to craft the findings of the study in the best possible way for the company.”

The body of literature available to the public, said the scientist, “is a biased sample of what companies want people to see.” The research scientist described “a culture of secrecy,” which makes it hard even for industry scientists tasked with ensuring drug safety to obtain the full datasets needed to genuinely understand a drug's risk–benefit profile.

## Conclusion

Whistleblowers have been compared to bees—they have just one sting to use and using it may lead to career suicide [11]. Many of the whistleblowers at the roundtable said they had experienced retaliation from their employers for raising concerns, but all had felt obligated to speak out about practices in medicine and medical research that they believe are risking the public's health or safety. Graham said he felt “trapped by the truth” and had to act. “There are bigger issues here,” said Kruszewski. “I felt right from the start [that] if I wallowed in self-pity about being fired and having my belongings piled in the gutter that I would never understand why all these things were happening. The bigger issue is that we've got people in the pharmaceutical industry and the health-care industry all acting in synchrony.”

Each of these whistleblowers, in very different ways—from making a satiric film to speaking out in Congress—has shone light on how this “synchrony” may be compromising the integrity of American medicine. We should not have to rely on medical whistleblowers to alert us to these fault lines. If we are to restore objectivity to drug development, prescribing, and safety monitoring, we must be willing to examine and change all of the institutions that allow this synchrony to occur.

## Abbreviation

FDA: Food and Drug Administration
